# Calcineurin regulates cyclin D1 stability through dephosphorylation at T286

**DOI:** 10.1038/s41598-019-48976-7

**Published:** 2019-09-04

**Authors:** Takahiro Goshima, Makoto Habara, Keisuke Maeda, Shunsuke Hanaki, Yoichi Kato, Midori Shimada

**Affiliations:** 1 Department of Cell Biology, Graduate School of Medical Sciences, Nagoya City University, 1 Kawasumi, Mizuho-cho, Mizuho-ku, Nagoya, Aichi 467-8601 Japan; 2 Department of Biochemistry, Joint Faculty of Veterinary Science, Yamaguchi University, 1677-1 Yoshida, Yamaguchi, 753-8511 Japan

**Keywords:** Cell growth, Phosphorylation

## Abstract

The Calcineurin/NFAT (nuclear factor of activated T cells) pathway plays an essential role in the tumorigenic and metastatic properties in breast cancer. The molecular mechanism of the antiproliferative effect of calcineurin inhibition, however, is poorly understood. We found that calcineurin inhibition delayed cell cycle progression at G1/S, and promoted cyclin D1 degradation by inhibiting dephosphorylation at T286. Importantly, overexpression of cyclin D1 partially rescued delayed G1/S progression, thereby revealing cyclin D1 as a key factor downstream of calcineurin inhibition. Cyclin D1 upregulation is observed in human invasive breast cancers, and our findings indicate that dysregulation of T286 phosphorylation could play a role in this phenomenon. We therefore propose that targeting site specific phosphorylation of cyclin D1 could be a potential strategy for clinical intervention of invasive breast cancer.

## Introduction

Intracellular calcium (Ca^2+^) regulates a number of diverse cellular processes including cell proliferation, development, motility, secretion, learning and memory^1^. Furthermore, Ca^2+^ and its intracellular receptor, calmodulin (CaM), are required for cell cycle, especially in G1 progression^2^. However, the essential targets of Ca^2+^/CaM-dependent pathways required for cell proliferation remain largely elusive.

One of the major regulators of intracellular Ca^2+^ signaling is calcineurin [also known as serine/threonine-protein phosphatase 2B (PP2B)], which is a calcium - CaM - dependent serine/threonine phosphatase. Calcineurin dephosphorylates the nuclear factor of activated T cells (NFATc) transcription factors, allowing these factors to enter the nucleus and promote gene transcription in T cells^3^. In addition to its well investigated role in T cells, interestingly, calcineurin/NFAT signaling is activated in colorectal and breast cancer^4–6^. Especially in estrogen receptor (ER)- progesterone receptor (PR)- HER2- triple-negative breast cancer (TNBC), it promotes migration and invasion *in vitro* and growth and metastasis *in vivo*.

Cyclin D1 is a key regulator of G1/S transition, and cell proliferation^7^. Consistent with this role, in cancer cells, this molecule acts as an oncogene by promoting tumorigenesis and metastasis. Indeed, the *CCND1* gene encoding cyclin D1 is amplified in 15% of breast tumors, with overexpression of cyclin D1 protein found in 50% of cases^8^. In the TNBC cell line MDA-MB-231, cyclin D1 regulates TGFβ-mediated tumor growth initiation^9^. Moreover, there is a positive correlation between cyclin D1 and ERα expression^10–12^. Consistent with this observation, overexpression of cyclin D1 promotes cell proliferation, while cyclin D1 knockdown reduces S phase cells in the ER positive invasive breast cancer cell line MCF7^13^.

Of note, previous studies have shown that calcineurin regulates cyclin D1 via multiple mechanisms. Indeed, cyclosporine A, which is an inhibitor of calcineurin, causes G1 arrest by suppressing cyclin D1, and by altering the function of cdk4 which is a key co-factor of cyclin D1^2^.

In a molecular level, the cyclin D1 protein is dynamically regulated by phosphorylation at T286. Previous studies have identified GSK3β, p38 and ERK2 as putative kinases for T286^14–18^. Importantly, T286 phosphorylation activates downstream cascades, for example binding of the SCF (Skp, Cullin, F-box containing) E3 ubiquitin ligase. Ubiquitination by SCF leads to proteasomal degradation^14^, and thereby regulates cellular level of cyclin D1.

The above model also indicates that dephosphorylation by a putative phosphatase should inhibit SCF binding, and prevent cyclin D1 degradation via ubiquitin-mediated proteasomal pathways. Intriguingly, previous studies showed that inhibition of type 2A phosphatase - such as PP2A, PP4 and PP6 - by low doses of calyculin A, enhanced T286 phosphorylation and subsequent proteasomal degradation of cyclin D1. Knockdown of these phosphatases, however, did not upregulate cyclin D1 phosphorylation, nor did it cause enhanced degradation of cyclin D1^19^; raising doubts about the proposed role of type 2A phosphatase for regulation of cyclin D1. Phosphatases that target phosphorylated T286, therefore, remain elusive.

As mentioned already, calcineurin possesses protein phosphatase activity, regulates cyclin D1 in the G1 phase. These observations indicate that calcineurin could be a potential phosphatase for cyclin D1. In this study, we therefore examined the molecular mechanism by which calcineurin regulates cyclin D1, and asked whether the same mechanism played a role in control cell cycle in invasive breast cancer cells.

Indeed, inhibition of calcineurin by FK506 or CN585 led to delay in G1/S progression, and induced cell death. This was associated with increased T286 phosphorylation, and enhanced proteasomal degradation of cyclin D1. Consistent with these results, ectopic expression of cyclin D1 partially reversed the delay in G1/S progression. In addition, knockdown of calcineurin A downregulated cyclin D1 protein level, accompanied with inhibition of cell growth. Finally, we found that calcineurin dephosphorylates cyclin D1 at T286 *in vitro*.

Taken together, our data therefore reveal a new mechanism by which calcineurin inhibits cyclin D1 degradation by dephosphorylation of the T286 residue, facilitating cell cycle progression and robust cell growth in invasive breast cancer cells. We propose that selective inhibition of calcineurin could be an effective method for treatment of breast cancer, and other types of cancer in which cyclin D1 is overexpressed.

## Results

### FK506 represses breast cancer cell growth

**Figure 1:**
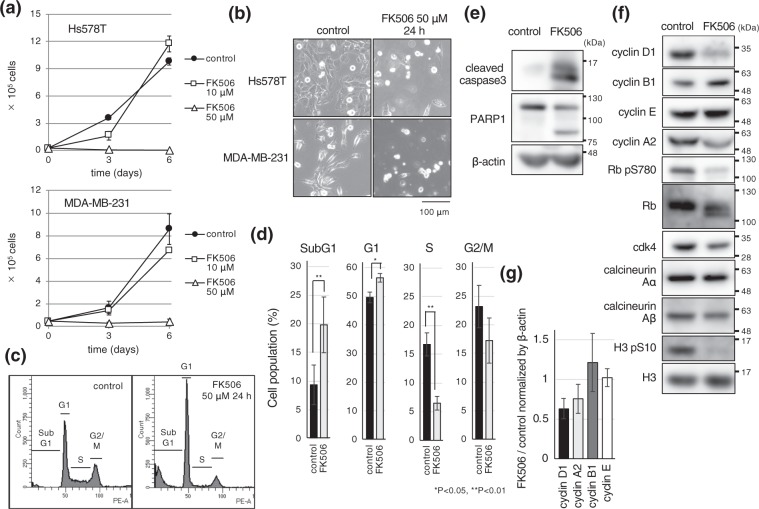
FK506 repressed breast cancer cell growth with decreased expression of cyclin D1. (**a**) Breast cancer cell lines, Hs578T and MDA-MB-231, were seeded and cultured in the presence of the indicated concentrations of FK506 or DMSO as a control. After 3 or 6 days, cells were collected and cell numbers were counted. Data are presented from three independent experiments. Error bars represent standard deviations (s.d.). (**b**) Hs578T and MDA-MB-231 cells were cultured in the presence of DMSO or FK506 (50 μM) for 24 h. Typical differential interference contrast images are shown. (**c**,**d**) Hs578T cells were cultured in the presence of FK506 (50 μM) or DMSO. After 24 h, cells were collected for FACS analysis to monitor cell cycle profiles. Data are presented from three independent experiments. Error bars represent standard deviations (s.d.). (**e**,**f**) Hs578T cells were treated with 50 μM FK506 or DMSO for 24 h. Cells were collected and total cell extracts were subjected to immunoblotting using the indicated antibodies. Blots have been cropped. Full uncropped blots are available in Supplemental Fig. S3. (**g**) Relative band intensity of cyclin D1, A2, B1 and E were normalized by β-actin and compared to the control.

Given that the calcineurin/NFAT pathway is activated in invasive breast cancer^4^, we investigated the effect of a calcineurin/NFAT inhibitor, FK506, on cell proliferation in breast cancer cell lines, namely, Hs578T and MDA-MB-231. FK506 (50 μM) efficiently inhibited cell growth (Fig. 1a), which was consistent with a previous report showing that FK506 induced apoptosis in Jurkat cells and other non-T cell lines^20–27^. Indeed, we observed that after 24 h treatment with FK506, cells became rounded and detached from culture dishes (Fig. 1b). FACS analysis revealed that FK506 led to an increase of sub-G1, and conversely, decrease of S phase cells (Fig. 1c,d). To confirm that FK506 promotes apoptosis, we examined two apoptotic markers, namely, caspase 3 and PARP1^28^. Both were efficiently cleaved (Fig. 1e), indicating that FK506 induced apoptosis in breast cancer cells, by inhibiting calcineurin.

### FK506 reduces cyclin D1 protein level and Rb phosphorylation

The role of different cyclins for cell cycle progression is well documented. Indeed, cyclins D and E regulate progression from the G1 to the S phase, by activating cyclin-dependent kinases. In addition, cyclin A2 is required for continuation of the S phase, and cyclin B1 for the G2/M phase^7^. We therefore investigated the expression of these key cyclins in calcineurin/NFAT inhibitor treated cells. Interestingly, cyclin D1 protein level decreased upon FK506 treatment (Fig. 1f,g). In addition, the protein level of cyclin A2 was also slightly reduced by FK506 treatment, whereas expressions of cyclin B1 and E were not significantly changed. Cyclin D1/cdk4 phosphorylates key regulators of the cell cycle, such as the retinoblastoma protein (Rb). Rb represses cell cycle, and thereby acts as a tumor suppressor. Phosphorylation at multiple sites by cyclin D1/cdk4, however, inhibits the anti-proliferative roles of Rb^29^. As expected, Rb phosphorylation (S780), it is a target of cyclin D1/cdk4, was decreased by FK506 treatment (Fig. 1f,g). Of note, FK506 did not affect the expression levels of calcineurin A isoforms (Fig. 1f).

### FK506 leads to delayed G1/S progression

**Figure 2:**
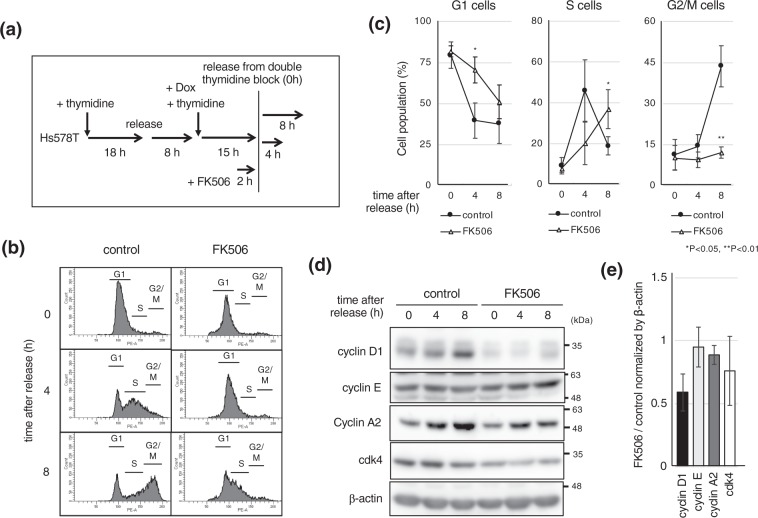
FK506 delayed cell cycle progression at G1/S. (**a**) The experimental strategy to synchronize Hs578T cells at the G1/S boundary using double thymidine block. Cells were treated with FK506 (50 μM) for 2 h before release from the thymidine block and released in the presence of FK506. (**b**,**c**) Cell cycle profiles at the indicated times were monitored by FACS analysis. Data are presented from three independent experiments. Error bars represent standard deviations (s.d.) (**d**) Cells were harvested at the indicated times after release, and expressions of  the indicated proteins were analyzed by immunoblotting. β-actin was used as a loading control. Blots have been cropped. Full uncropped blots are available in Supplemental Fig. S4. (**e**) Relative band intensity of indicated proteins at 0 h were quantified using imageJ and normalized by that of β-actin to compare with the control (0 h). Cyclin D1 data from three independent experiments and standard deviations (s.d.) are shown.

Given that cyclin D1 plays a role for cell growth we wondered if the downregulation of cyclin D1, by FK506 treatment, affected cell cycle progression. We synchronized cells at the G1/S boundary using double thymidine block (Fig. 2a), and examined the impact of FK506 treatment in cells. FK506 was added 2 h before the release from the thymidine block, and cells were cultured in the presence of FK506 after release. FACS analysis revealed that S phase progression was slower in FK506 treated cells, compared to the control (Fig. 2b,c). 8 h after release, 43.6 (±7.5) % of the control cells entered into the G2/M phase, whereas most of the cells treated with FK506 were still in the G1 or S phase, and only 12.1 (±2.1) % of the cells showed progression into the G2/M phase (Fig. 2c). FK506 treatment led to downregulation of cyclin D1 and A2, but had no effect on cyclin E (Fig. 2d,e). As reduction of cyclin D1 expression was prominent, we focused on this protein. Taken together, the above results indicate that FK506 suppresses breast cancer cell proliferation, accompanied with delayed G1/S progression, by repressing cyclin D1/cdk4.

### CN585, an inhibitor of calcineurin, also affects cell growth

**Figure 3:**
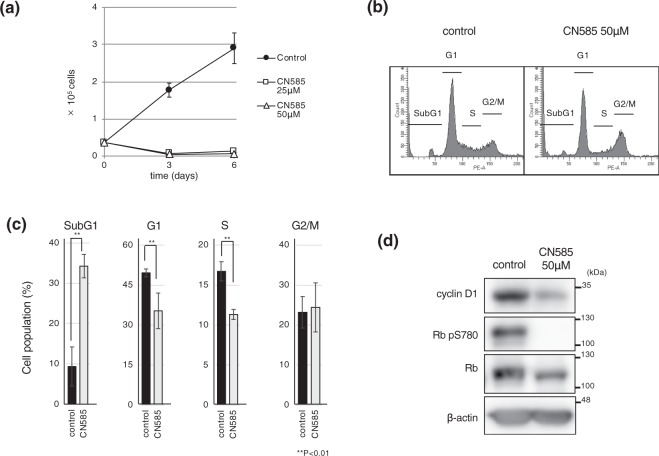
CN585 repressed breast cancer cell growth with decreased expression of cyclin D1. (**a**) Hs578T was seeded and cultured in the presence of CN585 (25 μM or 50 μM) or DMSO as a control. After 3 or 6 days, cells were collected and cell numbers were counted. Data are presented from three independent experiments. Error bars represent standard deviations (s.d.). (**b**,**c**) Hs578T cells were cultured in the presence of CN585 (50 μM) or DMSO. After 24 h, cells were collected for FACS analysis to monitor cell cycle profiles. Data are presented from three independent experiments. Error bars represent standard deviations (s.d.). (**d**) Hs578T cells were treated with CN585 or DMSO for 24 h and total cell extracts were analyzed by immunoblotting using the indicated antibodies. Blots have been cropped. Full uncropped blots are available in Supplemental Fig. S5.

Given that FK506 inhibits not only calcineurin but also FK506 binding proteins (FKBP)^30^, therefore we further treated cells with CN585, which is an additional inhibitor of calcineurin^31^. Consistent with the results obtained from FK506 treatment, administration of CN585 efficiently inhibited Hs578T cell growth (Fig. 3a). FACS analysis showed that CN585 treatment led to an increase of sub-G1, and a reciprocal decrease of S phase cells (Fig. 3b,c). In contrast to FK506 treatment (Fig. 1d), however, cells in the G1 phase did not increase. Calcineurin inhibition decreased cyclin D1 expression more prominently than cdk4 (Figs 1f and 2d). Therefore, we focused on cyclin D1 expression affected by CN585. Importantly, CN585 led to a decrease of cyclin D1 protein level, and downstream Rb pS780, which was reminiscent of FK506 treatment (Fig. 3d).

### CN585 delays G1/S progression

**Figure 4:**
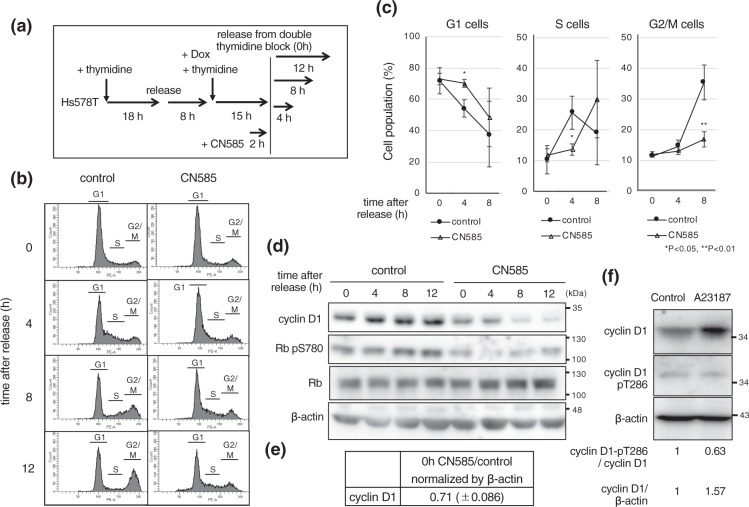
CN585 delayed cell cycle progression at G1/S. (**a**) The experimental strategy to synchronize Hs578T cells at the G1/S boundary using a double thymidine block. Cells were treated with CN585 (50 μM) for 2 h before release from the thymidine block and released in the presence of CN585. (**b**,**c**) The cells were synchronized and treated with CN585 as in (**a**), collected at indicated time points and monitored by FACS analysis. (**d**) Cells were cultured as in (**a**), and total cell extracts were analyzed by immunoblotting using the indicated antibodies. Blots have been cropped. Full uncropped blots are available in Supplemental Fig. S5. (**e**) Relative band intensity of cyclin D1 was quantified using imageJ and normalized by that of β-actin. Data was presented from three independent experiments and standard deviations (s.d.) were shown. (**f**) Hs578T cells were treated with 4 μM A23187 or DMSO for 30 min. Total cell extracts were analyzed by immunoblotting using the indicated antibodies. Relative band intensity of cyclin D1 pT286 or cyclin D1 was normalized by cyclin D1 or β-actin respectively, compared to the control, and quantified using imageJ.

To further probe the impact of CN585, we synchronized cells by double thymidine block (Fig. 4a). FACS analysis showed that the rate of S phase progression was slower in the cells treated with CN585, compared to the control (Fig. 4b,c). 8 h after release, 35.4 (±5.7) % of the control cells entered into the G2/M phase, while most of the cells treated with CN585 were still in the S phase; and 16.9 (±2.5) % of the cells showed progression into the G2/M phase (Fig. 4c). In addition, CN585 treatment downregulated cyclin D1 and also decreased Rb phosphorylation (Fig. 4d,e). These results indicate that CN585 causes delay in G1/S progression, similar to FK506. Importantly, we found that the activator of calcineurin by the calcium ionophore A23187 (causing influx of extracellular Ca^2+^)^32^ promoted dephosphorylation of cyclin D1-pT286 and increased the expression of cyclin D1 (Fig. 4f). These observations indicate that A23187 causes calcineurin activation, and as a result dephosphorylation of cyclin D1.

### Knockdown of calcineurin reduces cyclin D1 expression and population of S phase cells

**Figure 5:**
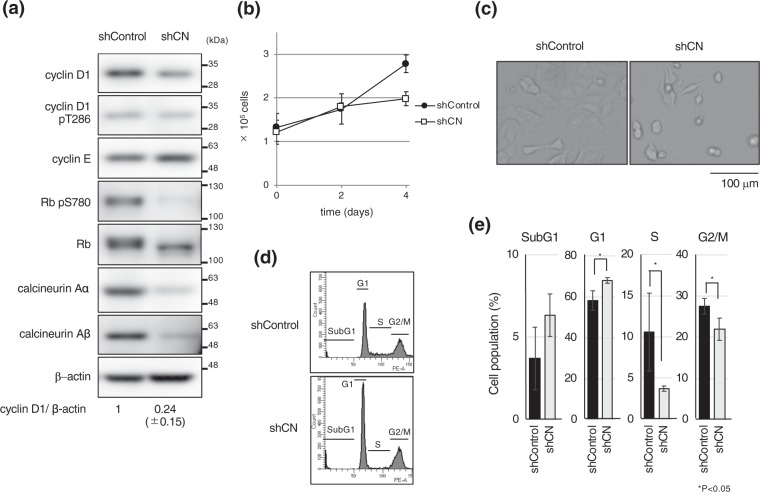
Calcineurin A knockdown similarly alters cyclin D1 expression and cell cycle. MCF7 cells were cultured in the presence of Dox for 3 days to knock down calcineurin Aα and Aβ (the catalytic subunit of calcineurin, α and β isoform) or Luciferase using tetracycline-inducible shRNA. (**a**) Cells were collected at 3 days after addition of Dox and total cell extracts were analyzed by immunoblotting using the indicated antibodies. Relative band intensity of cyclin D1 was quantified using imageJ and normalized by that of β-actin. Data are presented from three independent experiments. Blots have been cropped. Full uncropped blots are available in Supplemental Fig. S6. (**b**) Cells expressing shControl or shCalcineurin A were cultured in the presence of Dox and cell numbers were counted. Data are presented from three independent experiments. Error bars represent standard deviations (s.d.). (**c**) Typical differential interference contrast images at 3 days are shown. (**d**,**e**) Cells were collected after 3 days of culture in the presence of Dox and FACS analysis was performed. Data are presented from three independent experiments. Error bars represent standard deviations (s.d.).

To directly show that calcineurin is required for cyclin D1 expression, we knocked down the expression of two major isoforms of calcineurin A (Aα and Aβ) using a lentivirus-delivered shRNA technique. We found that cyclin D1, but not cyclin E, was reduced in calcineurin knockdown cells (Fig. 5a). As observed in FK506 or CN585 treated cells, Rb was dephosphorylated in calcineurin knockdown cells, confirming that cyclin D1/cdk4 was inactivated as a result of calcineurin depletion. Calcineurin knockdown led to repressed cell growth (Figs 5b,c and S1a,b), reduced number of S phase cells, and increased number of sub-G1 cells (Figs 5d,e and S1c); which are similar to the results obtained from the cells treated with FK506. Taken together, we concluded that calcineurin is required for cyclin D1 expression and proper cell cycle progression.

### Inhibition of cyclin D1 degradation partially rescues FK506 or CN585 induced delay in G1/S progression

**Figure 6:**
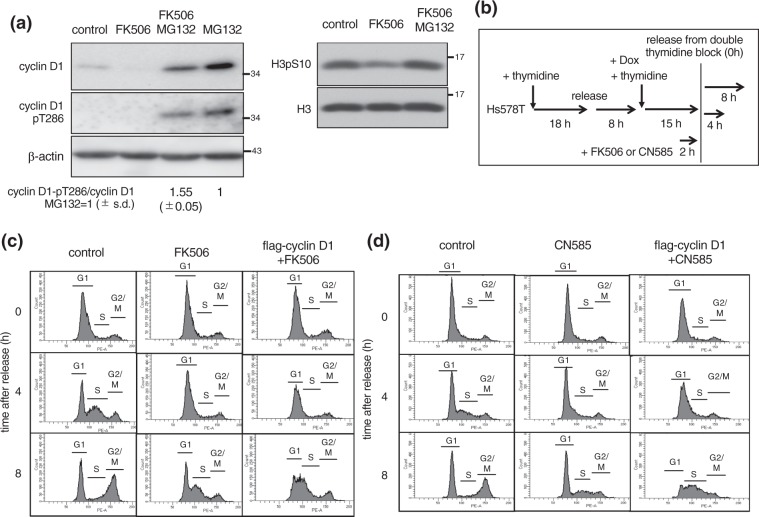
Overexpression of cyclin D1 partially rescued delayed G1/S progression. (**a,b**) Hs578T cells were treated with 50 μM FK506 and with or without 10 μM proteasome inhibitor MG132 for 2 h. Total cell extracts were analyzed by immunoblotting using the indicated antibodies. Relative band intensity of cyclin D1 pT286 was normalized by cyclin D1. Relative ratios of cyclin D1 pT286/cyclin D1 from three independent experiments are shown in the bottom. Blots have been cropped. Full uncropped blots are available in Supplemental Fig. S7. (**b**) Experimental strategy to synchronize Hs578T cells at the G1/S boundary using a double thymidine block, and for inducing flag-cyclin D1 expression. Cells were treated with FK506, CN585 or DMSO for 2 h prior to release. (**c**,**d**) Cell cycle profiles after release as in (**b**) were monitored by FACS analysis.

We asked if blocking cyclin D1 degradation, by inhibition of the ubiquitin-mediated proteasomal pathway, rescued the phenotypes induced by FK506. To this end, we treated the cells with FK506 with or without the proteasome inhibitor MG132 (Fig. 6a). Indeed, cells treated with MG132 showed reduced degradation (i.e. increased protein level) of cyclin D1. Next, we investigated if FK506 induced degradation of cyclin D1 through dephosphorylation. We found that T286-phosphorylation was increased by 1.55-fold in FK506 and MG132 treated cells, compared to only MG132 treated cells, indicating that FK506 inhibits dephosphorylation of cyclin D1, leading to degradation of cyclin D1. Of note, a similar response was seen in the MCF7 breast cancer cell line, upon FK506 and MG132 treatment (Supplemental Fig. S2a).

We examined if FK506 also regulated cyclin D1 at the transcriptional level. To this end, we examined the amount of cyclin D1 transcripts after 2 h and 6 h of FK506 treatment. At 2 h, there was a modest change in cyclin D1 transcription, which became more pronounced after 6 h (Supplemental Fig. S2b). These results suggest that FK506 regulates cyclin D1 levels by a two-pronged mechanism. Upon shorter incubation (2 h) of treatment, FK506 promotes mainly degradation of the cyclin D1 protein by phosphorylation at T286. However, upon longer incubation (6 h), FK506 leads to repression of cyclin D1 transcription. Consistent with these results, blocking cyclin D1 degradation by MG132 treatment rescued the delay in cell cycle progression, accompanied by increased phosphorylation of H3 on Ser10, which is a hallmark of mitotic cells (Fig. 6a).

We directly test the role of cyclin D1 degradation in G1/S delay, we overexpressed cyclin D1 in FK506 or CN585 treated cells (experimental strategy shown in Fig. 6b). Indeed, expression of cyclin D1 partially reversed the delay in G1/S progression (Fig. 6c,d). Taken together, these results indicate that delayed G1/S progression observed upon calcineurin inhibition is caused by decreased cyclin D1 levels.

### FK506 promotes phospho-T286 dependent cyclin D1 degradation

**Figure 7:**
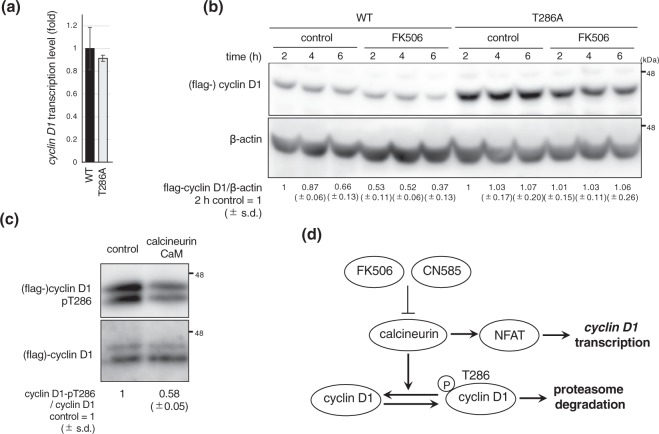
Dephosphorylation of cyclin D1 by calcineurin inhibited phosphodegron-mediated degradation by the proteasome. (**a**) Hs578T cells overexpressing flag-cyclin D1 WT or T286A were synchronized at G1 as in Fig. 6b and quantitative RT-PCR of *cyclin D1* was performed. Flag-cyclin D1 WT and T286A were tetracycline-inducible and cells were collected after 24 h from Dox addition. Data are presented from three independent experiments. Error bars represent standard deviations (s.d.). (**b**) Hs578T cells were treated with DMSO or FK506 for indicated times and cyclin D1 was analyzed by immunoblotting. β-actin was used as a loading control. Relative band intensity of cyclin D1 was normalized by β-actin, compared to the control cell groups (WT 2 h and T286A 2 h), and quantified using imageJ. (**c**) Phosphatase assay was performed with/without recombinant human calcineurin and calmodulin and reactions were followed by immunoblotting and probed with the indicated antibodies. Phosphorylated cyclin D1 was quantified and normalized relative to the total flag-cyclin D1. The results are expressed at the bottom of the panel as relative levels of cyclin D1-pT286 compared with negative control. Blots have been cropped. Full uncropped blots are available in Supplemental Fig. S8. (**d**) Proposed model of cyclin D1 expression mediated by calcineurin. In addition to transcriptional regulation via NFAT, our data indicates that calcineurin dephosphorylates cyclin D1 on T286, which inhibits cyclin D1 degradation.

To investigate the mechanism of cyclin D1 degradation upon calcineurin inhibition, we expressed WT, or a degradation resistant mutant (T286A)^14^, cyclin D1 in FK506 treated cells. We confirmed that both WT and T286A transcripts were expressed similarly (Fig. 7a). As expected, the WT protein was degraded in the presence of FK506, while the T286A mutant was stable (Fig. 7b). These data suggest that FK506 induces cyclin D1 degradation through phosphorylation at T286.

Finally, given that enhanced phosphorylation of cyclin D1 was observed in cells treated with FK506, we investigated whether calcineurin could dephosphorylate cyclin D1 *in vitro*. Indeed, purified recombinant calcineurin mixed with calmodulin was able to dephosphorylate T286 of cyclin D1, as detected by a phosphatase assay (Fig. 7c).

## Discussion

Several reports showed that calcineurin inhibitors such as FK506 or cyclosporine A induces apoptosis in cancer cells^20–27^. The effects of FK506 on cell cycle progression, however, have not been characterized yet. Here, we show that FK506 inhibits cell growth, characterized by reduced number of cells in the S phase; and induces cleavages of caspase 3 and PARP1 which are the hallmarks of apoptosis. These results are consistent with the observation in murine mast cells that FK506 led to a decrease in S phase cells^33^. Our study also revealed that FK506 downregulated cyclin D1, but not other cyclins, such as cyclin E, B1 (Figs 1f,g and 2d,e). By synchronizing cells at the G1/S boundary, we also found that treatment with FK506 delays G1/S progression with reduced expression of cyclin D1 (Fig. 2). Similar results were obtained with CN585, which is a specific inhibitor of calcineurin (Fig. 4). Cyclin A2 expression was slightly reduced by FK506 treatment. It is, however, important to note that calcineurin inhibition by cyclosporine A downregulated cyclin A and E in growth factor stimulated progression from G1 to S phase^34^. Therefore, calcineurin may also regulate cyclin A2 expression.

Cyclosporine A also causes delay of G1/S progression in various cell types^24–26,33,35–37^. Of note, previous studies indicate that the antiproliferative effects of cyclosporine A could be mediated by direct repression of cyclin D1 mRNA levels^2,35^. In a reciprocal manner, NFAT2, which is a key functional partner of calcineurin, activates cyclin D1 transcription^38,39^. These findings indicate a model by which calcineurin activates cyclin D1 transcription in association with NFAT2^3^. Consistent with the previous studies, we confirmed that the transcription of *cyclin D1* was inhibited at 6 h incubation of FK506 (Supplemental Fig. S2b). In the present study, we therefore opted for short (2 h) periods of treatment, to avoid the effects on cyclin D1 mRNA transcription caused by long-term administration of calcineurin inhibitors.

Interestingly, we found that FK506 inhibited dephosphorylation of cyclin D1, and as a result led to degradation of cyclin D1. As expected, treatment with MG132, an inhibitor of proteasome activity, reversed FK506-mediated depletion of cyclin D1 protein levels (Fig. 6a). As FK506 is an inhibitor of calcineurin, this finding indicates that calcineurin regulates cyclin D1 protein stability, and in turn cell cycle progression. Indeed, we confirmed that calcineurin A knockdown similarly alters cyclin D1 expression and cell cycle progression (Figs 5 and S1). In line with this model, cyclin D1 expression partially rescued delayed G1/S progression in FK506 or CN585 treated cells (Fig. 6c,d).

It is necessary to note that in addition to calcineurin, FK506 also inhibits FK506 binding proteins (FKBP)^30^. We therefore used an additional inhibitor of calcineurin, namely, CN585; to show that treatment with this drug caused similar repression of cyclin D1 protein levels, and delayed cell cycle progression. Reduction of cyclin D1 protein levels is therefore a direct effect of calcineurin inhibition via FK506 treatment.

In addition, we found that calcineurin inhibition led to impaired T286 dephosphorylation, and subsequent degradation of cyclin D1. Indeed, *in vitro* phosphatase assay revealed that calcineurin directly dephosphorylated cyclin D1. Thus, dephosphorylation of T286 by calcineurin likely inhibits proteasomal degradation of cyclin D1 (Fig. 7d).

In summary, we found that calcineurin inhibition promoted cyclin D1 degradation by impairing dephosphorylation, resulting in G1/S delay. Although prognostic impact of cyclin D1 overexpression on breast carcinoma is controversial, it is worthy to note that overexpression of cyclin D1 is detected in more than 50% of human invasive breast cancers^8^, and this may be partly due to deregulated proteasomal degradation. It has also been reported that the calcineurin/NFAT pathway is frequently activated in TNBC subgroups, and has an essential role in the tumorigenic and metastatic properties^4^.

Similarly, mutation on T286 has been reported in esophageal and endometrial cancers^40–43^. We therefore propose that therapeutic targeting of cyclin D1 could be useful for the prevention and treatment of cancers, such as invasive breast cancer, where cyclin D1 is overexpressed.

## Materials and Methods

### Cell culture and reagents

Hs578T cells and MDA-MB-231 cells were cultured in DMEM supplemented with 10% FBS. Cells were treated with FK506 (063–06191, Wako), MG132 (474790, Merch), thymidine (T1895, Sigma-Aldrich), CN585 (207003, Merck) and A23187 (C7522, Sigma-Aldrich). All reagents were dissolved in DMSO. FK506 was used at concentrations of 50 μM; MG132 and thymidine were used at 10 μM and 2 mM respectively. CN585 was used at 25 μM and 50 μM.

### Construction of expression vectors

We amplified and subcloned human *cyclin D1* (CCND1) (GenBank TM accession number NM_080720). Human *cyclin D1* genes (having no introns) were obtained by PCR amplification of human genomic DNA using primers that introduce EcoRI and XhoI sites at the flanking regions. (cyclin D1-F; AAAGAATTCATGGAACACCAGCTCCTG, cyclin D1-R; AAACTCGAGTCAGCTGTCCACGTCCC) pENTR1A-3 × flag-cyclin D1 was generated by ligation. pENTR1A-3 × flag-cyclin D1 vectors were incubated with CSIV-TRE-RfA-UbC-KT vectors and LR Clonase enzyme mix (Invitrogen) for 2 h at 25 °C, which produced CSIV-TRE-RfA-UbC-KT 3 × flag-cyclin D1.

### Mutagenesis

pENTR1A-3 × flag-cyclin D1 T286A was generated by KOD-Plus-Mutagenesis Kit. (SMK-101,2wq Toyobo).

(T286A-F; GCACCCACCGACGTGC, T286A-R; GCAAGCCAGGTCCACC).

### Lentivirus generation and infection

Lentivirus expressing the respective genes was generated by the co-transfection of 293T cells with pCMV-VSV-G-RSV-RevB (a gift from H. Miyoshi), pCAG-HIVgp (also a gift from H. Miyoshi), and the respective CSIV-TRE-RfA-UbC-KT using the calcium phosphate co-precipitation method. Cells infected with viruses were treated with 2 μg/ml puromycin (P7255, Sigma-Aldrich) or 10 μg/ml blasticidin (A1113903, Gibco) for 2 days. To express the inducible gene, doxycycline (Dox; D9891, Sigma-Aldrich) was added to the medium at a concentration of 1 μg/ml.

### Construction of shRNA

To generate lentivirus-based shRNA constructs, a 21 base shRNA-coding fragment with a ACGTGTGCTGTCCGT loop was introduced into pENTR4-H1 digested with BamHI/BglII. pENTR4-H1-shRNA vectors were incubated with CS-RfA-ETBsd vectors and LR Clonase enzyme mix (Invitrogen) for 2 h at 25 °C, which produced the CS-RfA-ETBsd-shRNA vector. The target sequences for lentivirus-based sh-RNAs were PPP3CA (calcineurin Aα)-1: GCGTATATGATGCCTGTATGG, PPP3CA-2: GCCAAGGGCTTAGACCGAATT, PPP3CB (calcineurin Aβ)-1: GCTCAAGATGCAGGCTATAGA, PPP3CB-2: GCAATTGGCAAGATGGCAAGA.

### Immunoblotting

Collected cells were washed with ice-cold PBS, suspended with sample buffer (2% SDS, 10% glycerol, 100 μM dithiothreitol, 0.1% bromophenol blue, and 50 mM Tris-HCl at pH 6.8) and were boiled for 5 min. Samples were used as the total cell lysate. Antibodies used in this study are listed in Table 1. Raw digital images were captured using Amersham Imager 600 (GE Healthcare) or ChemiDoc Imaging system (BioRAD). The bands of the target protein were quantified using imageJ and normalized by that of β-actin otherwise indicated. Three independent experiments were performed and the representative image was shown in figures.

**Table 1:** Antibody used in this study.

H3 pS10	06-570	Merck
H3	ab1791	abcam
cyclin A2	sc751	Santa Cruz Biotechnology
cyclin B1	sc752	Santa Cruz Biotechnology
cyclin D1	sc753	Santa Cruz Biotechnology
cyclin D1 pT286	cs3300	Cell Signaling Technology
cyclin E	sc481	Santa Cruz Biotechnology
cdk4	sc749	Santa Cruz Biotechnology
Rb pS780	cs9307	Cell Signaling Technology
Rb	BD554136	BD Biosciences
β-actin	ab6276	abcam
cleaved caspase-3	cs9661	Cell Signaling Technology
PARP1	sc8007	Santa Cruz Biotechnology
PPP3CA (calcineurin Aα)	sc17808	Santa Cruz Biotechnology
PPP3CB (calcineurin Aβ)	13340-1-AP	proteintech

### Cell cycle synchronization

To synchronize Hs578T cells at the G1/S boundary, cells were treated with 2 mM thymidine for 18 h and then released into S phase by washout of thymidine with PBS and the addition of medium. After 8 h of release, these cells were exposed to 2 mM thymidine for 15 h and released again.

### Cell cycle analysis

Cells were cultured and fixed with 70% ethanol. These were then washed once with PBS, treated with RNase, and stained with propidium iodide. Flow cytometry was performed using a FACS CANTO2 or FACS Verse flow cytometer (BD Biosciences). Cell cycle profile was analyzed using BD FACSDiva™ software (BD Biosciences). Three independent experiments were performed and representative data was shown in figures.

### Real-time PCR

Total RNA was extracted using ISOGEN II (311–07361, NIPPON GENE, Tokyo, Japan) according to the manufacturer’s protocol and was subjected to reverse transcription. A total of 50 ng RNA was reverse-transcribed with random primers using High Capacity cDNA Reverse Transcription Kit (4368814, ABI). Quantitative real-time PCR was performed using FastStart Universal SYBR Green Master (11226200, Roche) and an ABI7900HT real-time PCR system (Applied Biosystems). Expression levels were normalized to GAPDH. Primers used in real-time PCR were *cyclin D1*-F:GAAGCCCTGCTGGAGTCA, *cyclin D1-*R:CCAGGTCCACCTCCTCCT, *GAPDH*-F:GCCAATCTCAGTCCCTTCC and *GAPDH*-R: TAGTAGCCGGGCCCTACTTT.

### Phosphatase assay

Flag-cyclin D1 expressed in Hs578T cells was pull downed with anti-flag M2 affinity gel (A2220, Sigma-Aldrich) and eluted with FLAG-peptide (F4799, Sigma-Aldrich) in assay buffer (20 mM Tris, 10 mM MgCl_2_, 0.1 mM CaCl_2_, 1 mg/mL BSA, pH 7.5). Flag-cyclin D1 was incubated with or without recombinant human calcineurin (3160-CA, R&D systems) and calmodulin (208670, Merk) in assay buffer and incubated for 2 h at 37 °C.

### Statistical analysis

Differences between two data groups were compared with student’s t-test. Results were considered statistically significant at p-values *p < 0.05 and **p < 0.01.

## Supplementary information

Supplementary Figure

## Data Availability

The data that support the findings of this study are available from the corresponding author upon reasonable request.
