# Supplementary material for: Calcineurin regulates cyclin D1 stability through dephosphorylation at T286
Source: Sci Rep. 2019 Sep 4;9:12779. doi: 10.1038/s41598-019-48976-7 (PMC6726757; doi:10.1038/s41598-019-48976-7)
Supplement: Supplementary file 1 — Supplementary Figure [file 41598_2019_48976_MOESM1_ESM.pdf]

Supplementary information

Calcineurin regulates cyclin D1 stability through dephosphorylation at T286

Takahiro Goshima, Makoto Habara, Keisuke Maeda, Shunsuke Hanaki, Yoichi Kato
and Midori Shimada

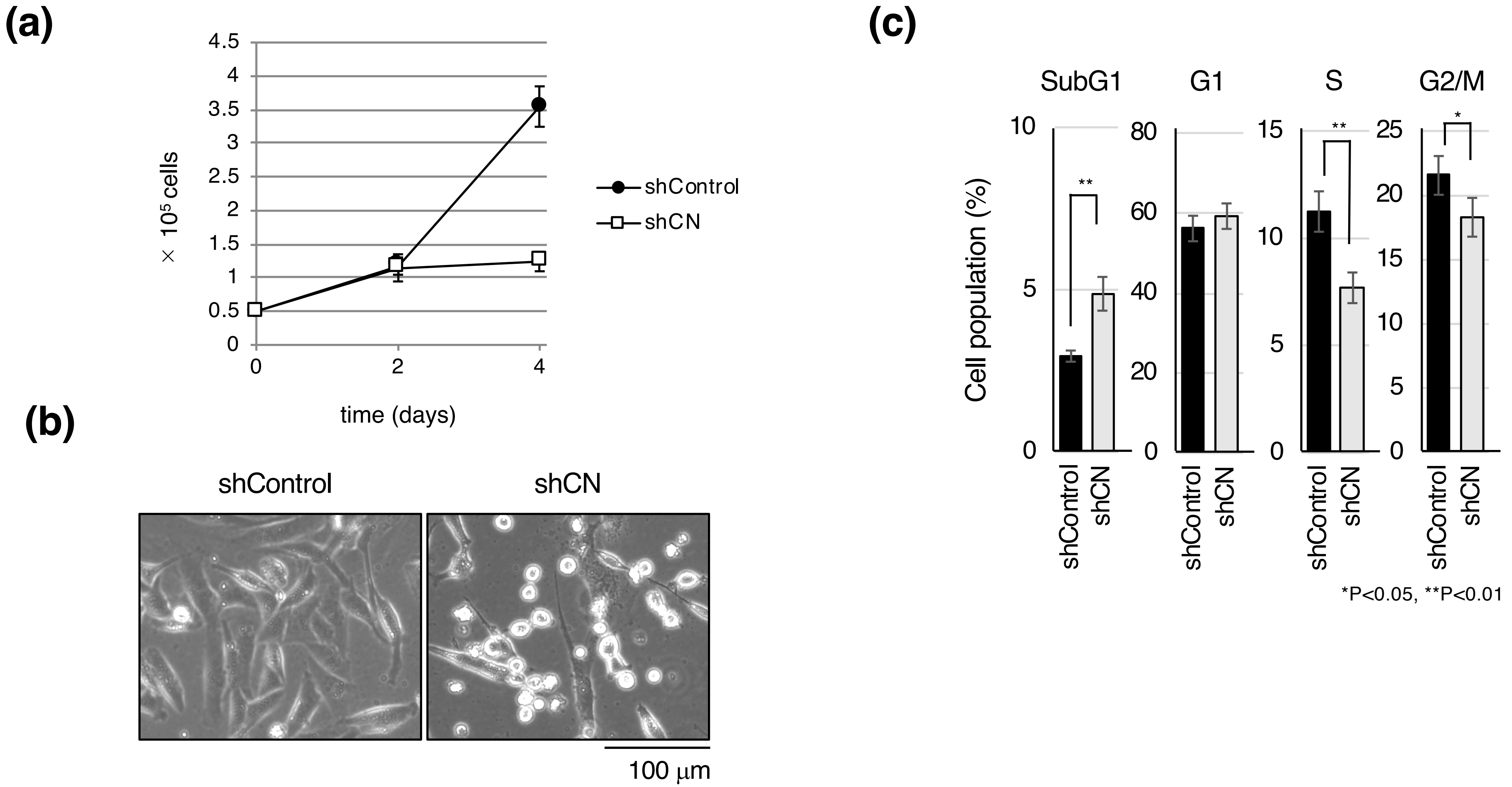

Supplemental Figure S1.

(a) MDA-MB-231 cells expressing shControl or shCalcineurin A were cultured in the presence of Dox and cell numbers were counted. Data are presented from three independent experiments. Error bars represent standard deviations (s.d.). (b) Typical differential interference contrast images at 3 days are shown. (c) Cells were collected for FACS analysis to monitor cell cycle profiles after 3 days of culture in the presence of Dox. Data are presented from three independent experiments. Error bars represent s.d.

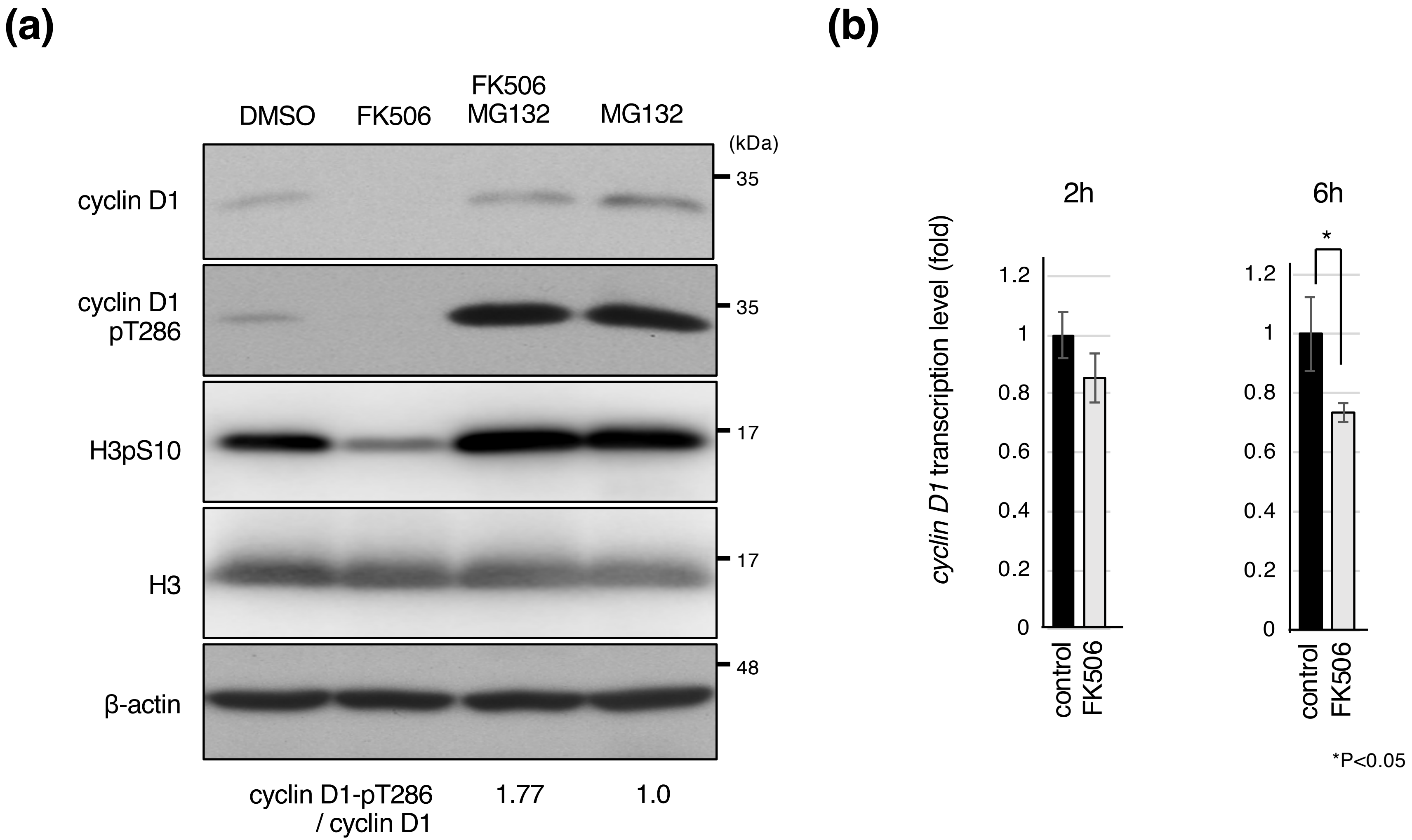

Supplemental Figure S2.

(a) MCF-7 cells were treated with 50 μM FK506 and with or without 10 μM proteasome inhibitor MG132 for 2 h. Expression level of cyclin D1 and cyclin D1 pT286 was detected by immunoblotting. β-actin was used as a loading control. Blots have been cropped. Full uncropped blots are available in Supplemental Figure S7. (b) Quantitative RT-PCR of *cyclin D1* was performed in Hs578T cells after indicated times of FK506 treatment. Data are presented from three independent experiments. Error bars represent s.d.

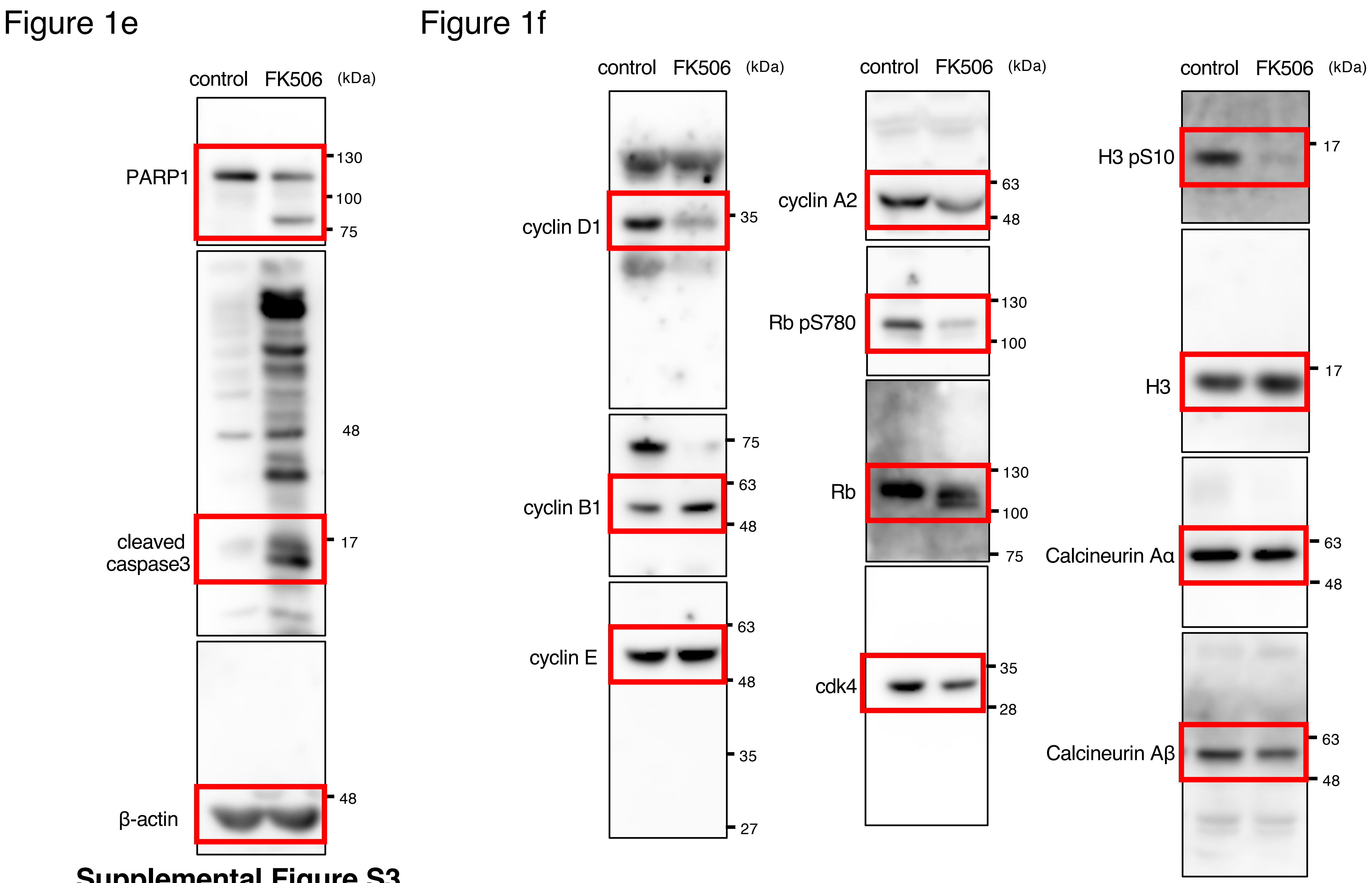

Supplemental Figure S3.
Full unedited images for Figure 1e and 1f are shown.

Figure 2d

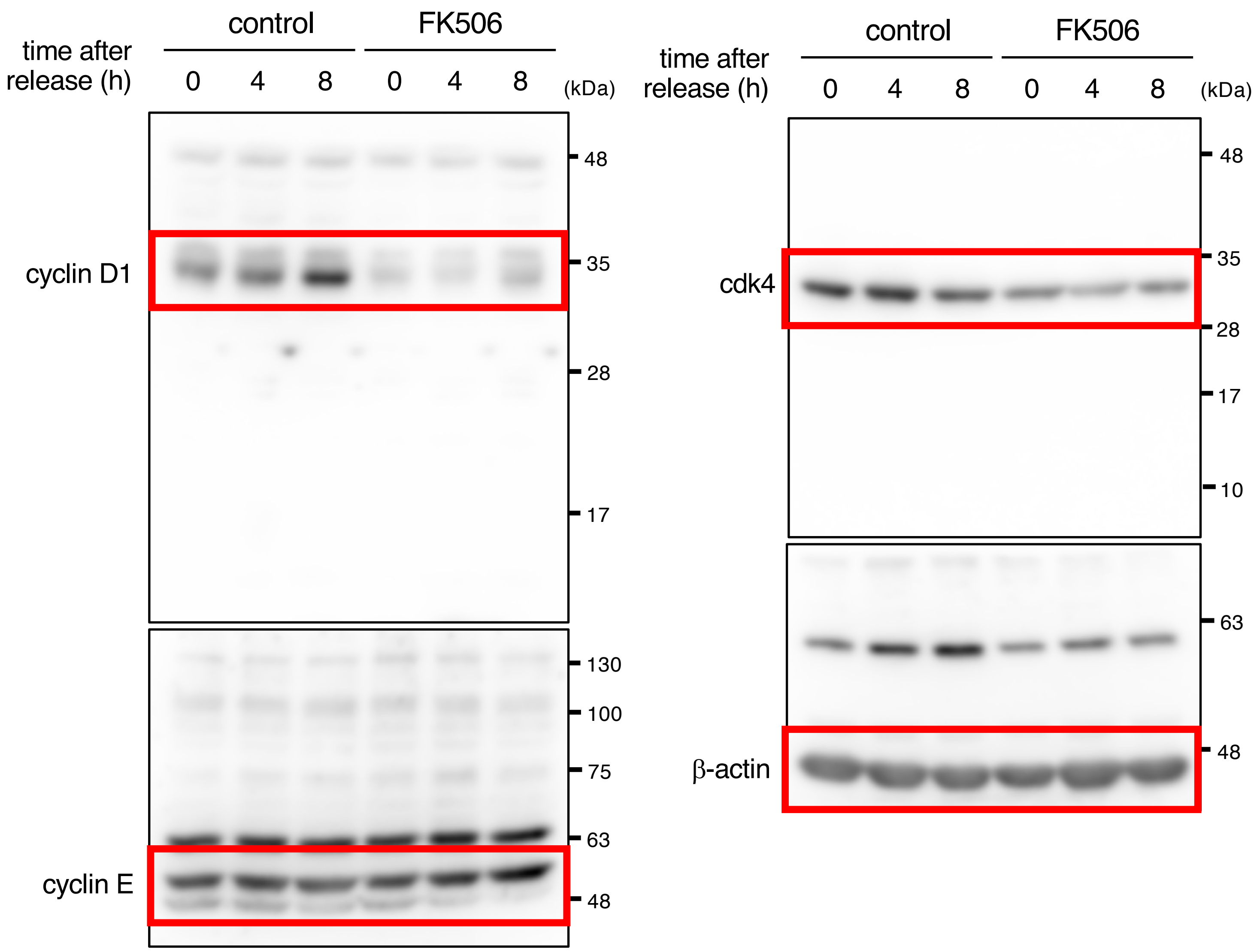

Supplemental Figure S4.
Full unedited images for Figure 2d are shown.

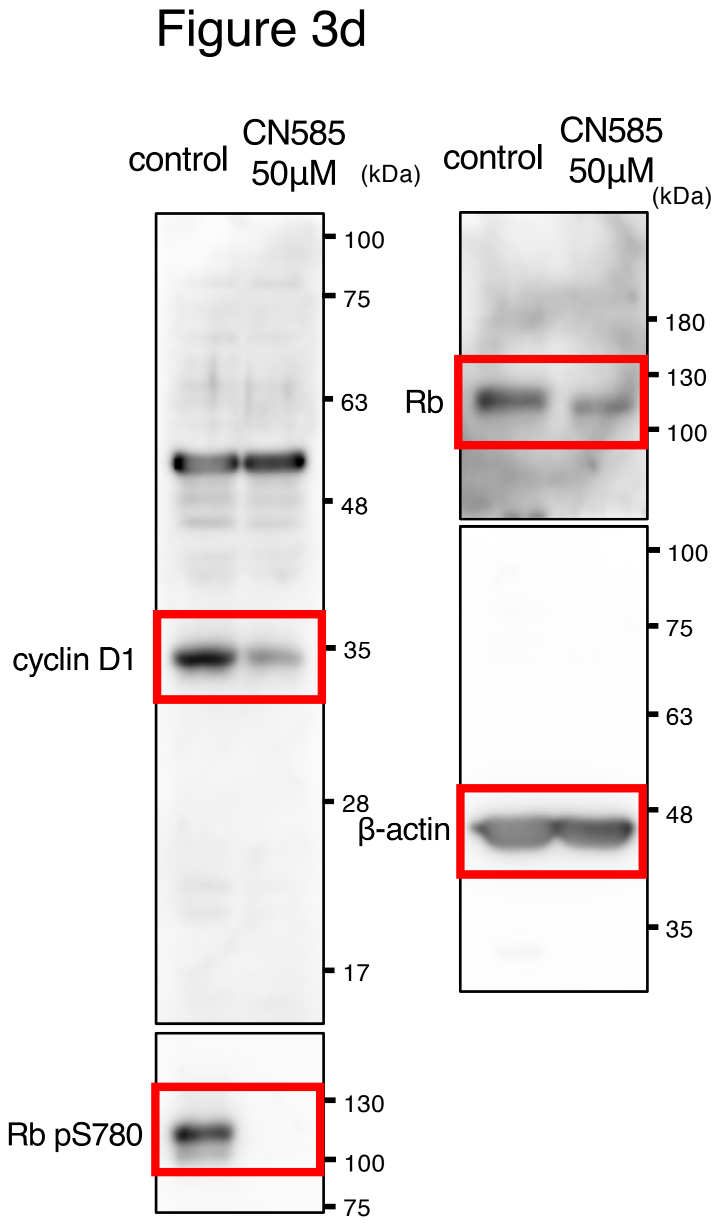

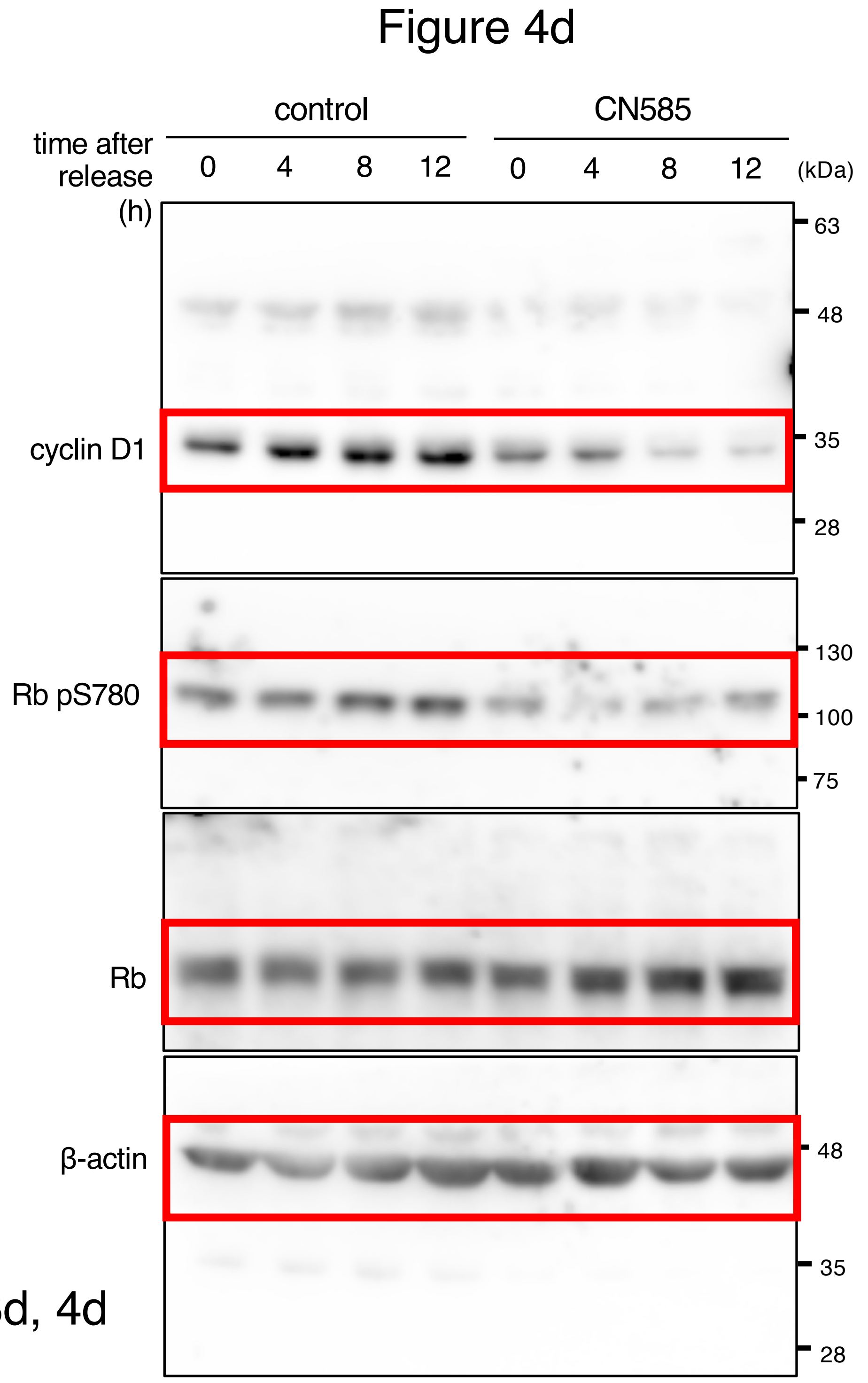

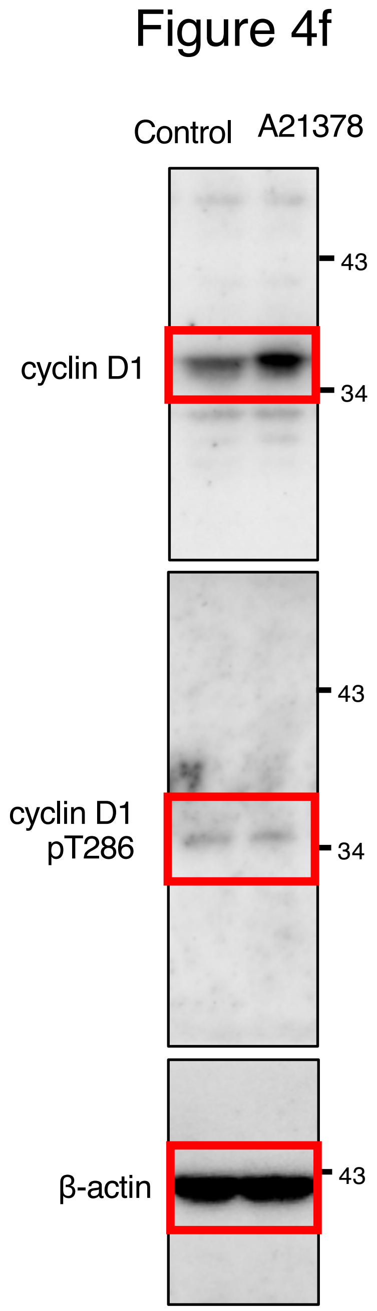

Supplemental Figure S5.
Full unedited images for Figure 3d, 4d and 4f are shown.

Figure 5a

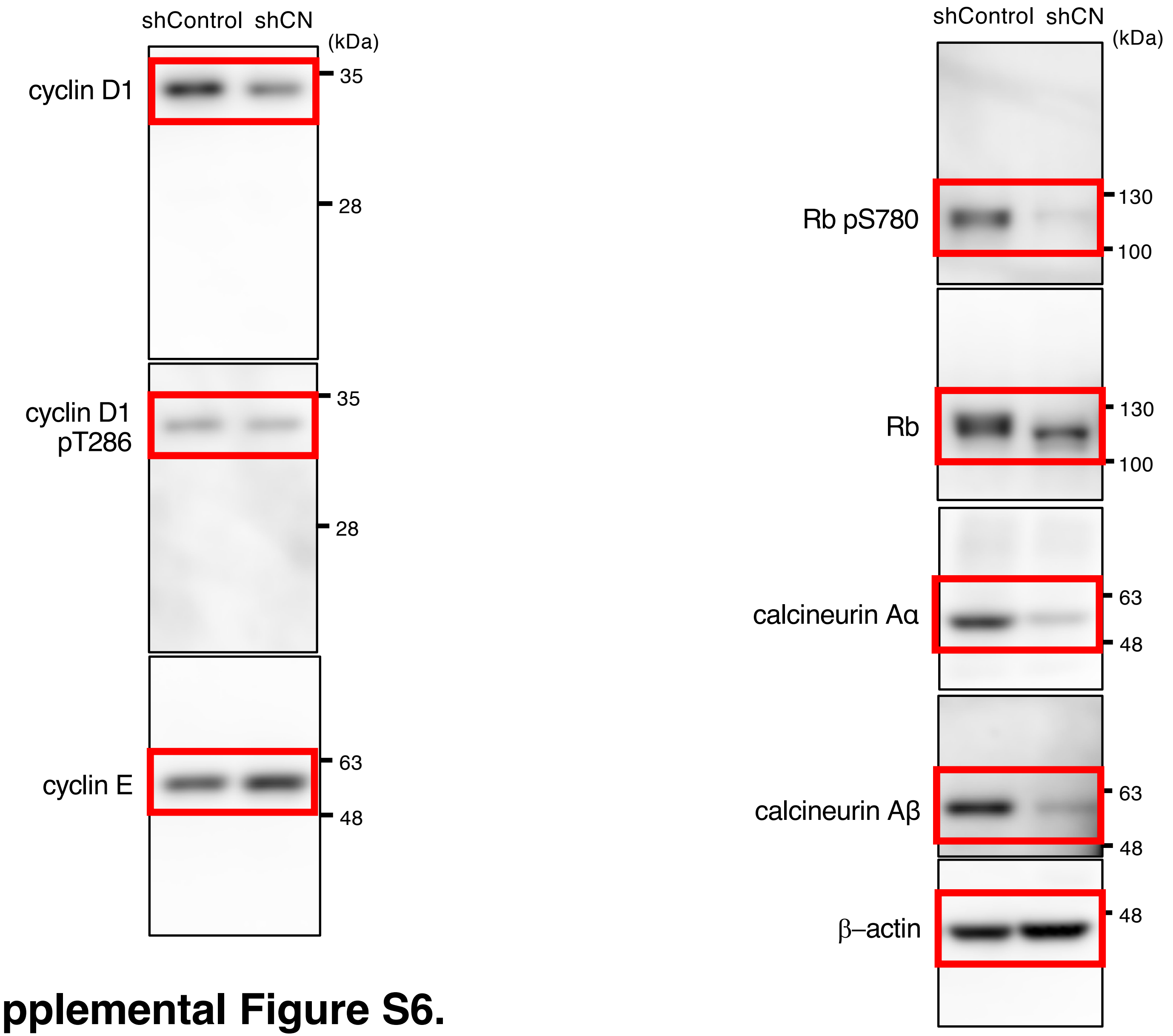

Supplemental Figure S6.
Full unedited images for Figure 5a are shown.

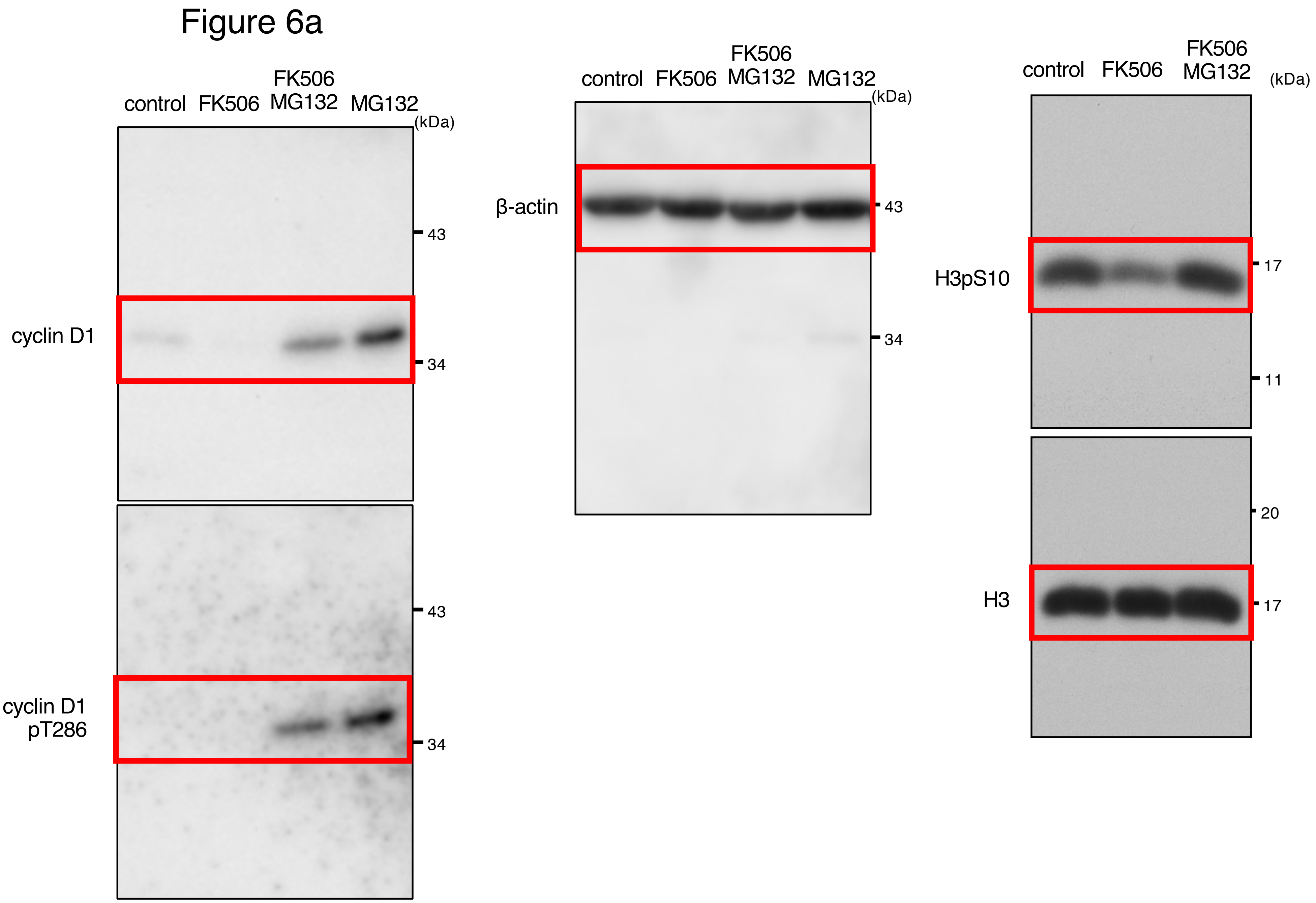

Supplemental Figure S7.
Full unedited images for Figure 6a are shown.

Figure 7b

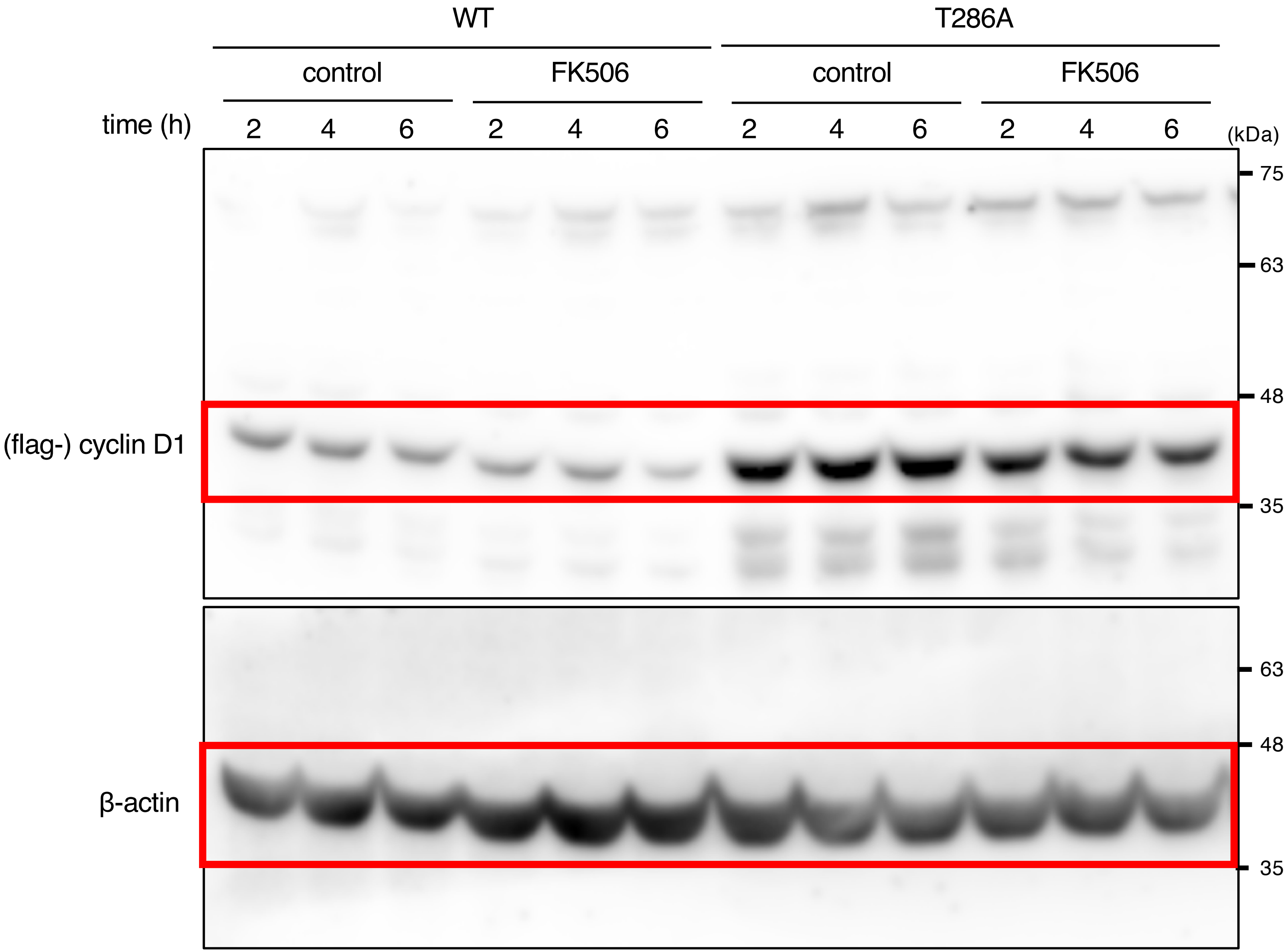

Figure 7c

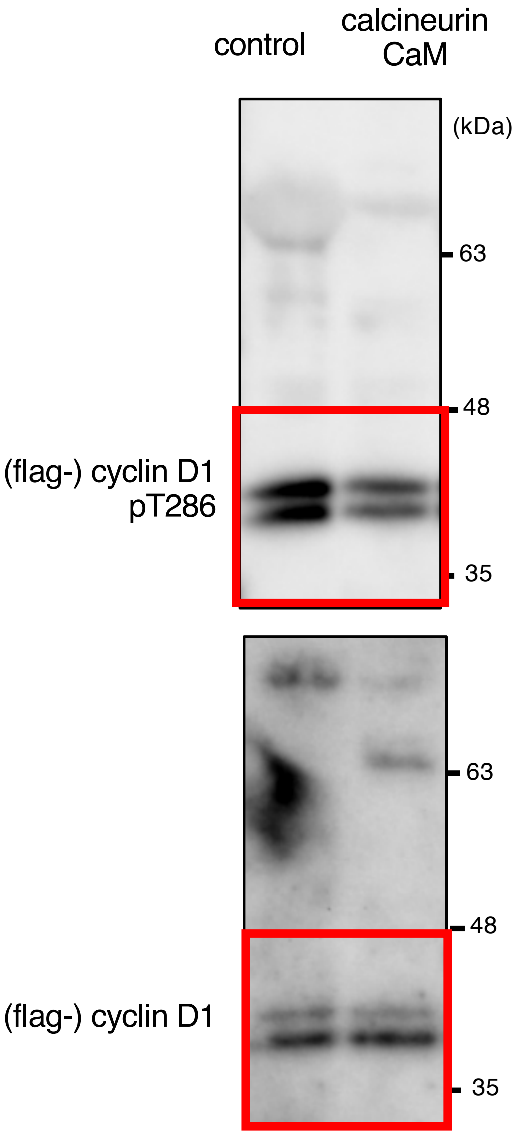

Supplemental Figure S8.
Full unedited images for Figure 7b and 7c are shown.

Figure S1

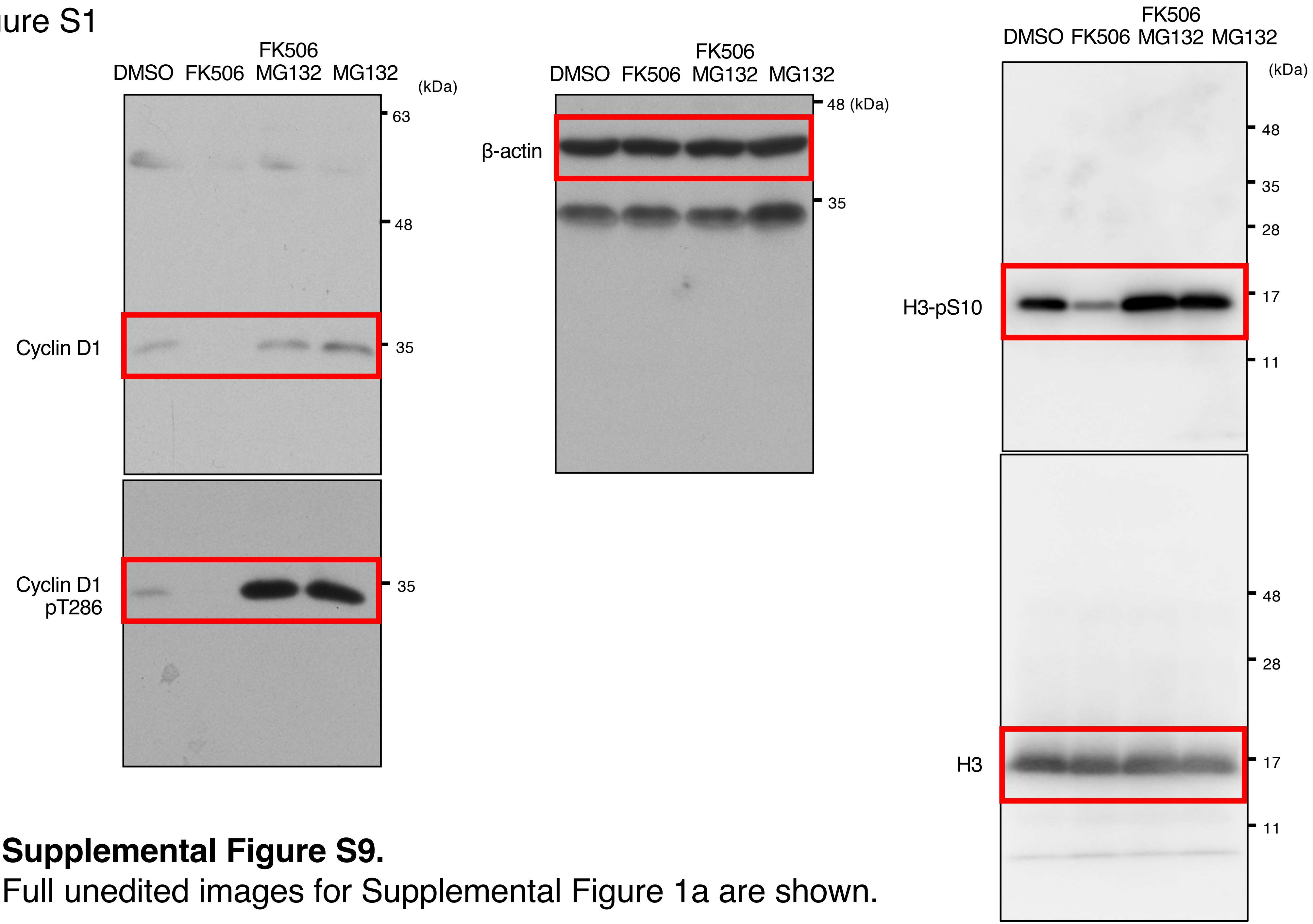

Supplemental Figure S9.
Full unedited images for Supplemental Figure 1a are shown.
